# Analysis of 20alpha-hydroxysteroid dehydrogenase expression in the corpus luteum of the buffalo cow: effect of prostaglandin F2-alpha treatment on circulating 20alpha-hydroxyprogesterone levels

**DOI:** 10.1186/1477-7827-11-111

**Published:** 2013-12-11

**Authors:** Tripathy Sudeshna, Kumarasamy Anand, Rudraiah Medhamurthy

**Affiliations:** 1 Department of Molecular Reproduction, Development and Genetics, Indian Institute of Science, Bangalore 560012, India

**Keywords:** Buffalo cow, Corpus luteum, PGF_2α_, P4, 20α-HSD, 20α-OHP, Nur77

## Abstract

**Background:**

During female reproductive cycles, a rapid fall in circulating progesterone (P4) levels is one of the earliest events that occur during induced luteolysis in mammals. In rodents, it is well recognized that during luteolysis, P4 is catabolized to its inactive metabolite, 20alpha-hydroxyprogesterone (20alpha-OHP) by the action of 20alpha-hydroxysteroid dehydrogenase (20alpha-HSD) enzyme and involves transcription factor, Nur77. Studies have been carried out to examine expression of 20alpha-HSD and its activity in the corpus luteum (CL) of buffalo cow.

**Methods:**

The expression of 20alpha-HSD across different bovine tissues along with CL was examined by qPCR analysis. Circulating P4 levels were monitored before and during PGF2alpha treatment. Expression of 20alpha-HSD and Nur77 mRNA was determined in CL at different time points post PGF2alpha treatment in buffalo cows. The chromatographic separation of P4 and its metabolite, 20alpha-OHP, in rat and buffalo cow serum samples were performed on reverse phase HPLC system. To further support the findings, 20alpha-HSD enzyme activity was quantitated in cytosolic fraction of CL of both rat and buffalo cow.

**Results:**

Circulating P4 concentration declined rapidly in response to PGF2alpha treatment. HPLC analysis of serum samples did not reveal changes in circulating 20alpha-OHP levels in buffalo cows but serum from pseudo pregnant rats receiving PGF2alpha treatment showed an increased 20alpha-OHP level at 24 h post treatment with accompanying decrease in P4 concentration. qPCR expression of 20alpha-HSD in CL from control and PGF2alpha-treated buffalo cows showed higher expression at 3 and 18 h post treatment, but its specific activity was not altered at different time points post PGF2alpha treatment. The Nur77 expression increased several fold 3 h post PGF2alpha treatment similar to the increased expression observed in the PGF2alpha-treated pseudo pregnant rats which perhaps suggest initiation of activation of apoptotic pathways in response to PGF2alpha treatment.

**Conclusions:**

The results taken together suggest that synthesis of P4 appears to be primarily affected by PGF2alpha treatment in buffalo cows in contrast to increased metabolism of P4 in rodents.

## Background

In rats during pregnancy, catabolism of progesterone (P_4_) to its inactive metabolite, 4-Pregnen-20α-ol-3-one i.e. 20α-hydroxyprogesterone (20α-OHP) has been suggested to be one of the key mechanisms for regulation of circulating P_4_ concentration both in maternal and fetal compartments [1-3]. The enzyme, 20α-hydroxysteroid dehydrogenase (20α-HSD), classified as one of the members of aldo-keto reductase superfamily is responsible for conversion of P_4_ into 20α-OHP [1]. In mice null for 20α-HSD gene, the length of estrous cycle and the duration of pseudo pregnancy and pregnancy periods were significantly prolonged although serum P_4_ levels decreased low enough for delivery of pups at term of pregnancy [4,5]. In pregnant goats, low concentration of P_4_ and high concentration of 20α-OHP in the fetal blood, while high concentration of P_4_ and low concentration of 20α-OHP in maternal blood have been reported [2]. In the baboon, the activity of 20α-HSD in placenta was observed to be higher with a corresponding increase in the concentration of 20α-OHP in the fetal compartment during late pregnancy [3]. In many of these species, the observation of increased 20α-OHP levels in the placenta is suggestive of regulation of P_4_ concentration by the feto-placental unit and/or parturition process. Since 20α-HSD is essential for conversion of P_4_ into 20α-OHP, it can be suggested that 20α-HSD expression in placenta plays an important role during fetal development and/or parturition process. However, induction of 20α-HSD expression in the corpus luteum (CL) is one of the striking features of luteolysis that occurs immediately prior to parturition and lactogenesis in pregnant rats [6,7].

During PGF_2α_-induced luteolysis, concomitant with the decreased P_4_ concentration, an increased concentration of 20α-OHP has been reported in pregnant rats [8]. Rat cDNA expression array analysis findings have provided evidence for convergence of opposing actions of prolactin and PGF_2α_ on 20α-HSD expression in the CL [9]. Furthermore, during PGF_2α_ treatment, an early association of increased expression of nerve growth factor-induced clone-B (NGFIB, also known as Nur77, NR4A1, among other designations) and 20α-HSD has been observed, that suggests participation of Nur77 in the induction of expression of 20α-HSD gene [8]. Nur77 which functions as transcription factor is a nuclear receptor protein belonging to steroid receptor superfamily and is suggested to play an important role in cell fate decisions [10]. Nur77 was originally characterized as immediate early response gene and has been shown to regulate expression of a number of steroidogenic genes in the ovary [11,12]. Also, Nur77 has been implicated as mediator of thymocyte and T-cell apoptosis [13,14]. Studies suggest that Nur77 induces apoptosis by activation of genes involving both extrinsic and intrinsic apoptotic pathways [15,16].

Despite extensive research, the cellular and molecular mechanisms involved in the PGF_2α_-induced luteal regression remains poorly understood. At present, with the exception of studies in rodents, reports of examination of 20α-HSD expression in CL of other species are sparse [17,18]. Moreover, whether P_4_ undergoes catabolism in the CL during spontaneous and PGF_2α_-induced luteolysis has not been reported in other species. It should be pointed out that the function of CL in bovine species unlike species such as primates is largely under the control of luteolytic factor, PGF_2α_. With a view to further gain insights into the PGF_2α_-induced luteolysis, several experiments were carried out in the buffalo cows with the following objectives: 1) To study 20α-HSD expression in various tissues including the CL of the buffalo cow, 2) To examine expression of Nur77, expression and activity of 20α-HSD during the PGF_2α_-induced luteolysis in the buffalo cow, and 3) To determine the concentration of 20α-OHP during PGF_2α_-induced luteolysis. The experiments involving well established rat model for PGF_2α_-induced 20α-HSD expression and activity were included for purposes of comparison with buffalo cow experiments.

## Methods

### Reagents

Juramate® (Cloprostenol sodium, the synthetic analogue of PGF_2α_) was purchased from Jurox, Australia. P_4_ (GDN#337) antisera was kindly provided by Prof. G.D. Niswender, Colorado State University, Fort Collins, CO. DyNAzyme™II DNA polymerase was obtained from Finnzymes, Espoo, Finland. Moloney murine lukemia virus (MMuLV) reverse transcriptase (Revert Aid™), RNase inhibitor (RNasein), 10 mM dNTP mix and 100 bp ladder were obtained from MBI Fermentas, Germany. NADP (disodium salt) and NADPH (tetra sodium salt) was obtained from HiMedia Laboratories Pvt. Ltd., Mumbai, India. Reference standards for 4-Pregnen-20α-ol-3-one (20α-OHP) and P_4_ were obtained from Sigma-Aldrich, Bangalore, India. Oligo dT and oligonucleotide primers were synthesized by Sigma-Genosys, Bangalore, India. The high performance liquid chromatography (HPLC) grade acetonitrile was obtained from Qualigens, Mumbai, India. All other reagents were purchased from Sigma-Aldrich, Bangalore, India or sourced through local suppliers.

### Animals, experimental protocol, blood and CL collection schedule

#### Experiments in buffalo cows

a. Collection of different organs for assessment of 20α-HSD mRNA expression

Non lactating adult buffalo cows (*Bubalus bubalis;* Surthi breed) aged 5–6 years with a known history of normal cyclicity were recruited for the study. Tissues such as spleen, brain, skeletal muscle, kidney, mammary gland, lung, heart, liver, myometrium and CL (n = 3/tissue) were collected to analyse the expression of 20α-HSD across different tissues.

b. Characterization of PGF_2α_ effects on CL function

The day of onset of estrus was designated as day 0 of estrous cycle. To verify the presence of functional CL, blood samples were collected on days 3 to 7 of the cycle for monitoring circulating P_4_ concentration. In this experiment, Juramate® (PGF_2α_) was administered 500 μg i.m., on day 11 of estrous cycle and CL was collected immediately before (0 h), 3, 6, 18 and 36 and 60 h post PGF_2α_ injection. Blood samples were collected immediately before (n = 9 animals) and at different time intervals (n = 6 animals/ time point) post PGF_2α_ injection for determining serum P_4_ levels. Ovaries containing CL (n = 3/time point) were collected post slaughter and washed in sterile ice cold PBS and transferred into Dulbecco’s Modified Eagles Medium supplemented with penicillin (500 U/ml) and streptomycin (50 μg/ml) and transported to the laboratory on ice within 30 min of collection. Under sterile conditions, CL was extirpated, cut into eight to twelve pieces, transferred to labelled cryovials, snap frozen in liquid nitrogen and stored at −70°C until analysis.

#### Experiment in rats

##### Effect of PGF_2α_ treatment on luteal function in rats

It is well documented that PGF_2α_ treatment increases 20α-HSD expression in the CL and circulating 20α-OHP in pseudo pregnant rats [8,19]. We utilized pseudo pregnant rat model system to serve as reference (with regard to post PGF_2α_ treatment related rise in 20α-HSD expression and 20α-OHP concentration) for PGF_2α_ studies in buffalo cows. Three month old adult female rats (Wistar strain) were housed in a controlled environment and kept under a photoperiod of 12 h light and 12 h of darkness cycle with *ad libitum* access to food and water. Pseudo pregnancy was induced in female rats by cohabitation with vasectomised male rats on the afternoon of proestrus. Following cohabitation, female rats were examined for the presence of vaginal plug and/or subjected to screening of vaginal smears daily for the extension of the diestrus period. The presence of vaginal plug and/or upon confirmation of day 1 of continuous diestrus (observed for 3 consecutive days) following cohabitation with vasectomised male rats was designated as day 1 of pseudo pregnancy. The status of pseudo pregnancy was further confirmed by determining the presence of higher (>50 ng/ml) circulating serum P_4_ concentration on day 5 of pseudo pregnancy. On day 8 of pseudo pregnancy, rats were injected i.p. with PBS (control) or 10 μg/100 μl of Juramate® (PGF_2α_). Blood (n = 5 animals/time point) and CL (n = 5 animals/time point) were collected before and 24 h post treatments.

All procedures in animals were approved by the Institutional Animal Ethics Committee, Indian Institute of Science, Bangalore, India.

### Hormone assays

Serum P_4_ concentrations were determined by specific radioimmunoassay as reported previously [20]. The sensitivity of the assay was 0.1 ng/ml and the inter- and intra- assay coefficients of variation were <10%.

### RNA isolation

Total RNA was extracted from control and PGF_2α_ treated samples using Tri^®^ Reagent according to the manufacturer’s recommendations, as reported previously [20]. RNA was quantitated spectrophotometrically using ND-1000 (NanoDrop, Thermo Scientific, Wilmington, DE, USA). The quality and quantity of RNA were determined by electrophoresis on a 2% (w/v) formaldehyde agarose gel along with RNA samples of known concentration and A_260_: A_280_ ratio was >1.8.

### Semi quantitative RT-PCR

Semi quantitative RT-PCR analysis for 20α-HSD was carried out as described previously from the laboratory [20]. L19 expression was used to check for the efficiency of RT-PCR. The primers used for 20α-HSD gene were F:5′-CTGTAACCAGGTCGAATGTCAC-3′ and R:5′-GGGTAGTTCGGGTTCACCC-3′; and for L19 were F:5′-CCACATGTATCACAGCCTGTAC-3′ and R:5′-CTTGGTCTTAGACCTGCGG-3′. Primers were designed from recently reported cattle sequences submitted by Naidansuren et al., 2011 [17] [GenBank: GU064907] using Primer Express™ version 2.0 (Applied Biosystems, Foster City, CA, USA) spanning the exon-exon junctions. PCR products were resolved on 2% Tris- acetate-EDTA agarose gels containing ethidium bromide (0.5 μg/ml), and photographed under UV light and analysed using GBox chemi-HR16, gel documentation system (Synoptics Ltd, Cambridge, UK). The amplified PCR product was eluted and cloned into pGEM-T easy vector system I, sequenced and the nucleotide analysis revealed 71% homology with bovine placental and ovary 20α-HSD sequence [17].

### Quantitative real time PCR (qPCR)

The analysis was carried out as described previously from the laboratory [21]. The cDNA samples equivalent to 10 ng of total RNA were subjected to validation analysis on Applied Biosystems 7500 Fast Real Time PCR system with SDS v 1.4 program employing Power SYBR green 2X PCR master mix. The following primers were used for analysis, for 20α-HSD gene, F:5′-CTGTAACCAGGTCGAATGTCAC-3′ and R:5′-GGGTAGTTCGGGTTCACCC-3′; for Nur77 gene, F:5′-CTTCTTCAAGCGCACAGTGCAG-3′ and R: 5′-CTGTCTGTCCGGACAACTTCCTTC-3′ and for L19 gene, F:5′-CCACATGTATCACAGCCTGTAC-3′ and R:5′-CTTGGTCTTAGACCTGCGG-3′. Primers were designed using cattle sequences submitted at NCBI and ENSEMBL using Primer Express™ version 2.0 (Applied Biosystems, Foster City, CA, USA). The primers were designed to cover the exon- exon junctions. Real time PCR efficiencies were acquired by amplification of a standard dilution series (with 10 fold differences) in the Applied Biosystems 7500 Fast Real time PCR system with SDS v 1.4 program employing Power SYBR Green 2X PCR mix. The corresponding efficiencies (E) for 20α-HSD and Nur77 were calculated according to the equation: E = 10^[−1/slope]^ -1 [22] and an efficiency of >90% was obtained for both. Analysis of expression of each gene included a no template control (NTC) and generation of a dissociation curve. Expression levels of the genes validated were normalized by using L19 expression levels as calibrator (internal control) for each cDNA sample. The relative expression and fold change in gene expression was determined using ΔC_t_ and ΔΔC_t_ method, respectively.

Relative expression = 2^-ΔCt^ and fold change = 2^-ΔΔCt^, where C_t_ = Threshold cycle i.e. the cycle number at which the relative fluorescence of test samples increases above the background fluorescence, ΔC_t_ = [C_t_ gene of interest (unknown sample) - C_t_ of L19 (unknown sample)] and ΔΔC_t_ = [C_t_ gene of interest (unknown sample) - C_t_ of L19 (unknown sample)] - [C_t_ gene of interest (calibrator sample) - C_t_ of L19 (calibrator sample)]. PCR for each sample was set up in duplicates and the average C_t_ value was used in the ΔΔC_t_ equation.

### HPLC analysis

#### HPLC unit

The chromatographic separation of P_4_ and its metabolite, 20α-OHP was performed on reverse phase HPLC system (Agilent 1200). Samples were injected via thermostated autosampler. The stationary phase was a Zorbax Eclipse Plus C18 5 μm column (4.6 X 250 mm) comprising of dense monolayer of dimethyl-n-octadecylsilane stationary phase with improved ultrahigh purity Zorbax Rx-SIL porous silica support. The thermostatted column compartment was used at an ambient temperature of 25°C. The readings at 245 nm were taken using variable UV wavelength detector. The mobile phase was a mixture of water (pH 3.4) and acetonitrile with gradient elution from 20 to 66% acetonitrile in 9 min (held for 3 min), then from 66 to 100% acetonitrile in 22 min. Standards for P_4_ and 20α-OHP were run on HPLC to determine the elution time separately, as well as, together.

#### Standard and sample preparation and extraction

For HPLC analysis, known concentration of P_4_ and 20α-OHP standards were diluted in steroid free serum. To remove steroids, 10 ml of bullock serum was treated with 0.5 g of activated charcoal and stirred for 2 h at 4°C. The slurry was centrifuged at 1750 X g for 10 min. The clear supernatant was collected and stored as 1–2 ml aliquots at −20°C.

The lipid extraction from serum samples was carried out by addition of methanol-diethyl ether mixture. For rat serum extraction (n = 5/time point), 500 μl of serum was mixed with 50 μl methanol and 5 ml diethyl ether, vortexed manually for 2 min and solvents containing lipids were separated after precipitating aqueous phase in liquid nitrogen and evaporating the solvent on a 37°C water bath. After repeating the procedure two more times, the extracted lipid was reconstituted in 10% acetonitrile. For bovine serum (n = 5/time point) lipid extraction, same procedure as used for rat serum was followed but with 2.5 ml serum volume. The samples were run on the HPLC column as mentioned earlier. The run was analysed drawing chromatograms using the Agilent Chemstation software and the runs were compared with P_4_ and 20α-OHP standards.

### Preparation of CL tissue cytosolic fraction

All procedures were performed at 4^o^C. Frozen CL tissues (10–15 mg wet weight) from rat and buffalo cows were homogenized in 500 μl of potassium phosphate buffer (5 mM, pH 7.0) containing 1 mM EDTA, 1 mM dithiothreitol and 10% glycerol. Protease inhibitors, 1 mM phenylmethanesulfonyl fluoride and 20 μg of leupeptin/ml and 40 μg of aprotenin/ml were used. The homogenate was centrifuged at 10,500 X g for 90 min. The supernatant was used as the cytosolic fraction.

### Measurement of luteal 20α-HSD activity

The activity of 20α-HSD was determined by the method of Wiest et al., 1968 [6] with a few modifications. The assay medium was Tris–HCl buffer solution (0.1 mM, pH 8.0) containing 30 μM 20α-OHP, 300 μM NADP, 1 mM EDTA, 5 mM dithiothreitol and 3% ethanol for sterol solubilisation; dithiothreitol and NADP were added immediately before use. The enzyme reaction was initiated at 37°C by adding 12.5 μl sample into the assay medium with rapid mixing. The OD values were recorded spectrophotometrically at 340 nm for 3 min. For sample blank, the cytosolic fraction was mixed with reaction buffer and OD values were recorded. The change in the concentration of NADPH formed in samples was calculated from the NADPH standard graphs. The enzyme activity was defined as the amount of enzyme that could induce 1 nmol NADPH min^-1^ mg^-1^ protein at 37°C.

### Statistical analysis

Where applicable, data were expressed as mean ± SEM. The arbitrary densitometric units were represented as relative mRNA expression after dividing the band intensity for L19 of the corresponding sample. Comparisons between mean of two groups were carried out using a non-parametric test, Mann–Whitney test, without assuming the Gaussian distribution. For multiple comparisons, the data were analyzed by one way ANOVA, followed by the Newman-Keuls multiple comparison test (PRISM Graph Pad, version 5; Graph Pad Software, Inc., San Diego, CA). A p-value of <0.05 was considered to be significant.

## Results

### Expression of 20α-HSD in various tissues

**Figure 1:**
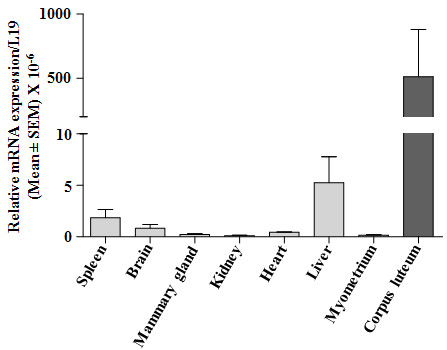
**Quantitative real time PCR expression of 20α-HSD mRNA in different tissues of buffalo cows.** 20α-HSD expression was normalized to L19 and the 20α-HSD mRNA expression is represented as relative expression level.

The qPCR expression of 20α-HSD mRNA was determined in various tissues of the buffalo cow and the results are presented in Figure 1. The mRNA expression was high in the CL and the expression was also detectable in spleen, brain and liver. However, the expression was low in mammary gland, kidney, heart and myometrium (Figure 1). In lung and skeletal muscle tissues, expression was undetectable (data not shown).

### Effects of PGF_2α_ treatment on circulating P_4_ levels, luteal expression of 20α-HSD and Nur77 in the buffalo cow

**Figure 2:**
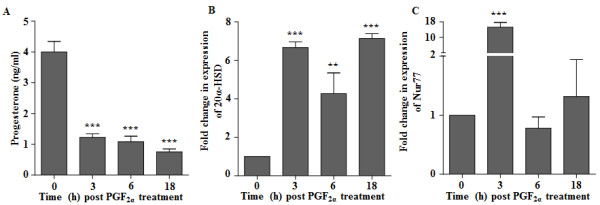
**Effect of PGF**_**2α **_**treatment on serum progesterone, luteal 20α-HSD and Nur77 mRNA expressions.** Buffalo cows received intramuscular injection of 500 μg of PGF_2α_ on day 11 of estrous cycle and blood and luteal tissue samples were collected at 0, 3, 6 and 18 h post PGF_2α_ treatment. **(A)** Mean (±SEM) circulating serum progesterone (P_4_) concentration immediately before (0 h) and after (3, 6 and 18 h) the PGF_2α_ treatment in buffalo cows, (***p < 0.001). **(B)** qPCR expression of 20α-HSD mRNA levels in the CL from buffalo cows collected at 0, 3, 6 and 18 h post PGF_2α_ treatment with the expression normalized with L19 mRNA. **(C)** qPCR expression of Nur77 mRNA levels in the CL from buffalo cows collected at 0, 3, 6 and 18 h post PGF_2α_ treatment and the expression was normalized with L19 mRNA. The results are shown as fold changes of mRNA expression compared with that at 0 h PGF_2α_. Bar represents mean ± SEM, n = 3, ***p < 0.001 and **p < 0.01 versus control (0 h).

Circulating P_4_ concentration in buffalo cows on day 11 of estrous cycle immediately before PGF_2α_ injection was 4.0 ± 0.34 ng/ml, and the concentrations were 1.23 ± 0.12, 1.09 ± 0.18 and 0.76 ± 0.09 ng/ml at 3, 6 and 18 h post treatment, respectively (Figure 2A). A significant (p < 0.001) decrease in P_4_ concentration was observed within 3 h post treatment and the concentrations further declined at 6 and 18 h time points. The fold change in expression of 20α-HSD mRNA in CL collected from control and PGF_2α_ treated animals are presented in Figure 2B. The 20α-HSD mRNA expression was 4–7 fold higher after PGF_2α_ treatment (Figure 2B). qPCR expression of Nur77 was >15 fold higher at 3 h post PGF_2α_ injection, however, the expression at other time points post PGF_2α_ injection was not significantly different from CL of PGF_2α_ untreated buffalo cows i.e. time 0 time point (Figure 2C).

### Analysis of P_4_ and 20α-OHP concentrations in biological samples by HPLC

**Figure 3:**
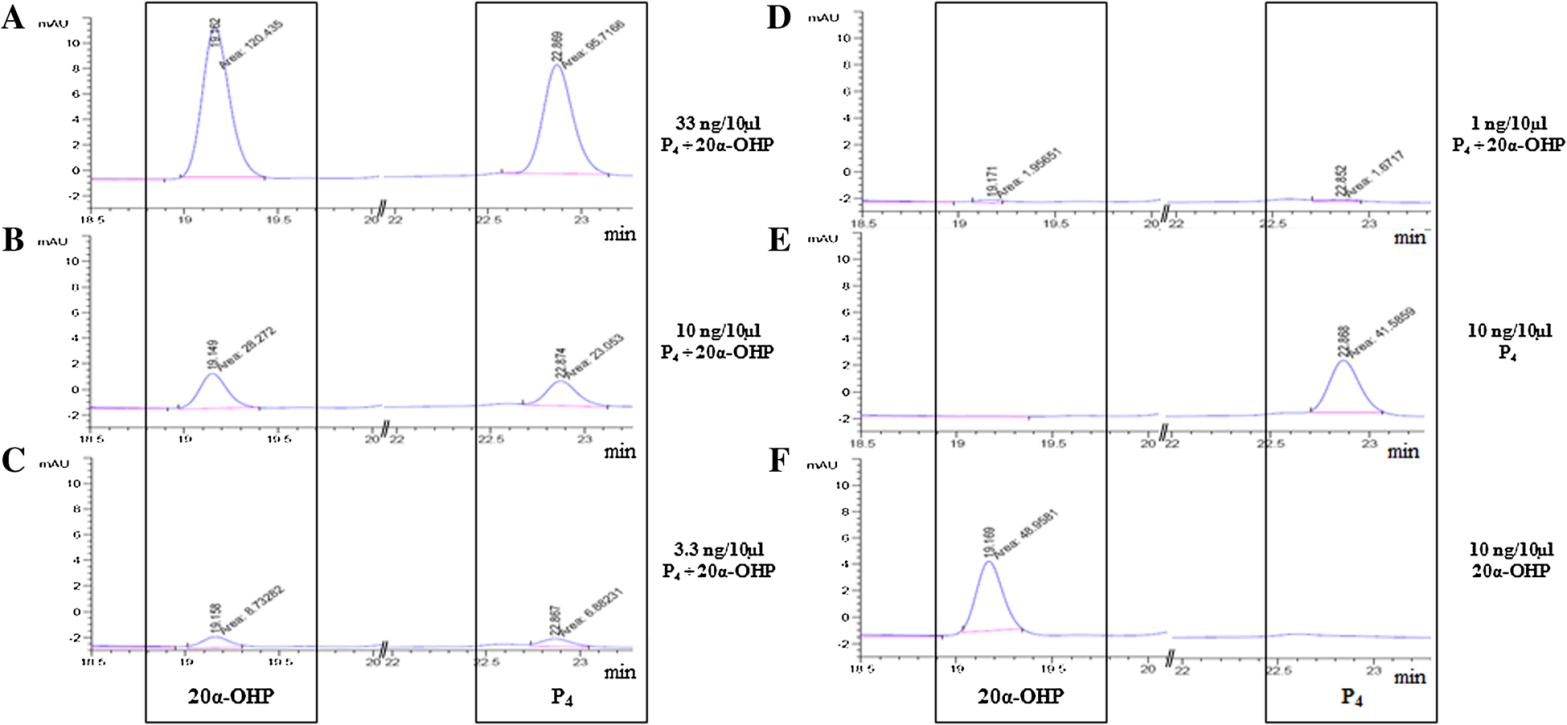
**Chromatograms of 20α-OHP and P**_**4 **_**standards.** Different concentrations of 20α-OHP and P_4_, 33, 10, 3.33 and 1 ng/10 μl **(A-D**, respectively**)**; P_4_, 10 ng/10 μl **(E)** and 20α-OHP, 10 ng/10 μl **(F)** were prepared and run on a Zorbax eclipse C18 column. The mobile phase comprised of acidified water: acetonitrile and sample injection volume of 10 μl was used. The retention time for 20α-OHP (19.1 min) and P_4_ (22.8 min) is indicated on each peak. Chromatograms of standards used for each 20α-OHP and P_4_ are boxed separately. The area under each peak corresponds to respective standard concentration.

After performing standardization of various parameters including standardization of the appropriate injection volume (10 μl) for determining the minimum detectable steroid concentration (2 ng/10 μl) and retention time (19.1 min for 20α-OHP and 22.8 min for P_4_, chromatograms not shown), known standards of varied concentrations of P_4_ and 20α-OHP either alone or after mixing both of them were run on a Zorbax eclipse Plus C18 column. The chromatogram patterns for a range of concentrations of mixture of P_4_ and 20α-OHP standards are shown in Figure 3. The area under peak (AUP) for each steroid was calculated and the data is presented in Table 1. The chromatogram patterns for fixed concentration of each steroid was also generated in order to rule out that the chromatogram pattern generated in mixture of two steroids was not different compared to pattern when fixed concentration of steroid was run (Figure 3). The representative chromatogram shown in Figure 3, shows an AUP of 120.44, 28.27, 8.73 and 1.96 units for 33, 10, 3.33 and 1 ng/10 μl (Figure 3A-D) of 20α-OHP, respectively. Further, an AUP of 95.72, 23.05, 6.89 and 1.67 units for 33, 10, 3.33 and 1 ng/10 μl (Figure 3A-D) is observed for P_4_, respectively.

**Table 1:** **HPLC analysis for different concentrations of 20α-OHP and P**_**4 **_**expressed as area under peak**

**Standards (ng/10 μl)**	**33**	**10**	**3.3**	**1**
*20α-OHP*	119.15 ± 5.22	35.71 ± 4.68	15.64 ± 6.99	4.45 ± 1.99
*P*_*4*_	102.48 ± 4.72	33.69 ± 4.49	14.51 ± 6.49	5.03 ± 2.25

Value represents mean ± SEM, in units.

**Figure 4:**
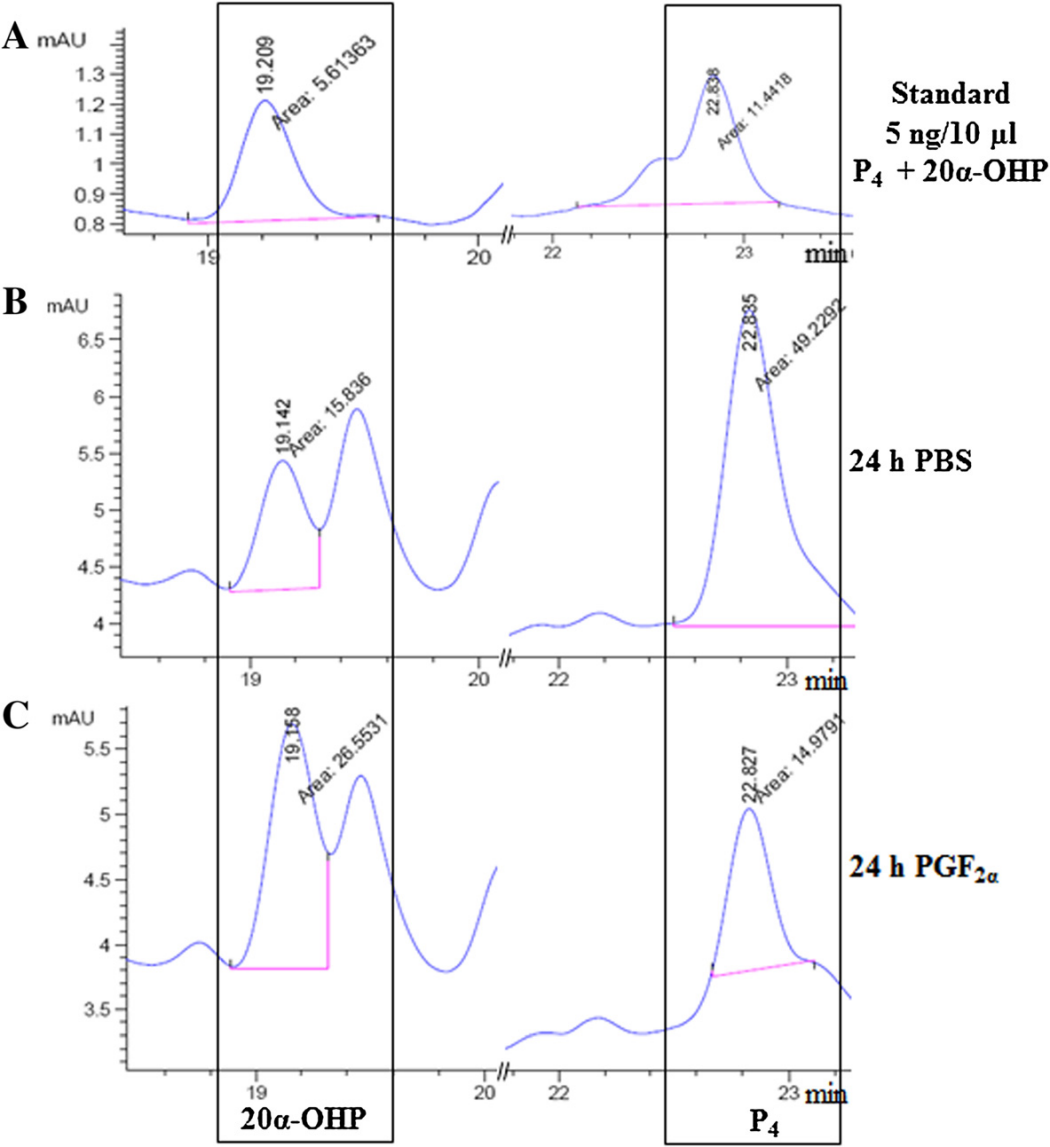
**Chromatograms of 20α-OHP and P**_**4 **_**in serum samples from pseudo pregnant rats. (A)** A concentration of 5 ng each of 20α-OHP and P_4_ was used during HPLC run. The peak for 20α-OHP and P_4_ were noted down at 19.1 and 22.8 min, respectively. **(B)** and **(C)** Chromatogram profiles of extracted serum samples collected from pseudo pregnant rats, 24 h treatment with PBS (control) or PGF_2α_ (treatment) are shown. The peaks of 20α-OHP and P_4_ were indicated at the respective run times for each steroid.

The profile for each steroid was determined on HPLC column for serum samples collected from rats 24 h after PBS (vehicle) or PGF_2α_ injection (Figure 4B and C) and the aggregate values for AUP is represented in Table 2.The AUP for 20α-OHP in serum was significantly increased (p < 0.05) in PGF_2α_ treated rats compared to PBS treated rats. On the other hand, the AUP for P_4_ peak was significantly decreased (p < 0.01) in serum from PGF_2α_ treated rats compared to serum from PBS treated rats.

**Table 2:** **HPLC analysis of 20α-OHP and P**_**4 **_**expressed as area under peak in rats and buffalo cows**

**Treatments**	**Rat**		**Buffalo cow**	
	**24 h PBS**	**24 h PGF**_**2α**_	**0 h PGF**_**2α**_	**18 h PGF**_**2α**_
*20α-OHP*	22.79 ± 3.58^a^	33.59 ± 2.99^b^	ND	ND
*P*_*4*_	55.89 ± 3.58^c^	23.09 ± 3.37^d^	22.02 ± 3.14^x^	13.18 ± 1.25^y^

Value represents mean ± SEM; ND = Non-detectable; p < 0.05 (a vs b, x vs y) and p < 0.01 (c vs d).

**Figure 5:**
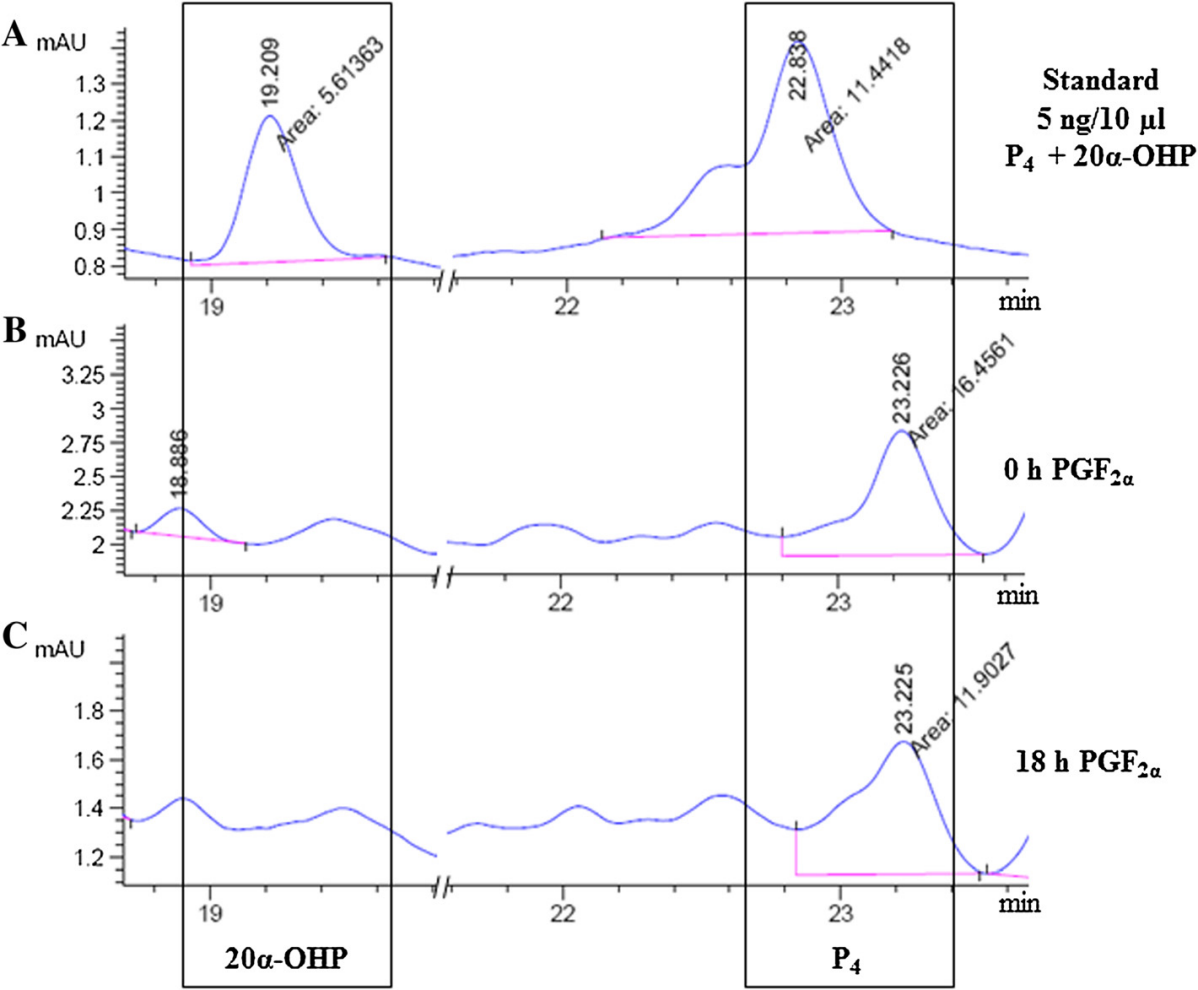
**Chromatograms of 20α-OHP and P**_**4 **_**in buffalo cow serum samples before and after PGF**_**2α **_**treatment. (A)** The standard mixture of steroids comprising of 5 ng each of 20α-OHP and P_4_ was used during HPLC run. The peak for 20α-OHP and P_4_ were noted down at 19.1 and 22.8 min, respectively. Chromatogram showing steroids extracted from serum collected at 0 h **(B)** and 18 h **(C)** post PGF_2α_ treatment in the buffalo cow are shown.

Similar to HPLC analysis of samples from rats, serum samples from buffalo cows receiving no treatment (0 h) and from animals receiving PGF_2α_ injection at 18 h time point were subjected to chromatographic analysis and a representative chromatogram pattern is presented in Figure 5. The sum total result of AUP values is represented in Table 2. The mixture of steroids at a concentration of 5 ng/10 μl for each HPLC run was analysed under identical HPLC conditions as shown in Figures 4A and 5A. The AUP for P_4_ peak significantly decreased (p < 0.05) in serum from 18 h post PGF_2α_ injected buffalo cows compared to serum of untreated buffalo cows (time 0 h) on day 11 of the estrous cycle (Figure 5B and C).

### Determination of luteal 20α-HSD activity in CL of pseudo pregnant rats and buffalo cows after PGF_2α_ treatment

**Figure 6:**
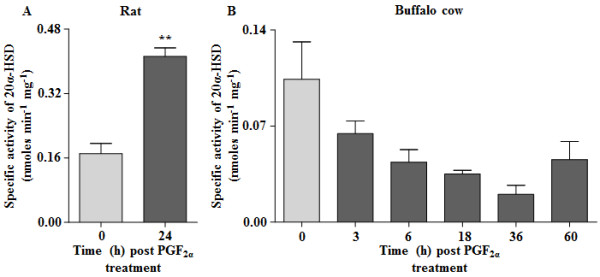
**Activity of cytosolic 20α-HSD in luteal tissue of pseudo pregnant rats and buffalo cows.** The enzyme activity is expressed as nmoles NADPH min^-1^ mg^-1^ with 1 unit of activity defined as the amount of enzyme that can induce formation of 1 nmole NADP min^-1^ mg^-1^ extract used at 37 ^o^C. **(A)** Specific activity of 20α-HSD at 0 and 24 h post PGF_2α_ treated pseudo pregnant rat CL cytosolic extract. **(B)** Specific activity of 20α-HSD at 0, 3, 6, 18, 36 and 60 h post PGF_2α_ treated buffalo CL cytosolic extract. The cytosolic extract from the luteal tissue collected without PGF_2α_ treatment (0 h) was designated as control. Each bar represents mean ± SEM, n = 5/time point for pseudo pregnant rats and n = 3/time point for buffalo cows, **p < 0.01, 0 h versus different time points.

Figure 6 shows the 20α-HSD activity both in rat and buffalo cow CL cytosolic fractions. The 20α-HSD specific activity (Figure 6A) was significantly higher (p < 0.01) in luteal tissue from PGF_2α_ treated rats compared to PBS treated rats (0.412 ± 0.02 vs 0.171 ± 0.03 nmoles min^-1^ mg^-1^ for CL from animals receiving PGF_2α_ and PBS treatment, respectively). On the other hand, examination of specific activity of 20α-HSD in cytosolic fractions of CL from PGF_2α_-treated buffalo cows at various time points did not change and tended to be lower from 0 h time point (Figure 6B).

## Discussion

Corpus luteum is a transient endocrine structure formed from the ovarian follicle after ovulation. Through biosynthesis and secretion of P_4_, it plays a pivotal role in the control of reproduction in mammals. The precise timing of expression of various enzymes/proteins required for synthesis and metabolism of P_4_ constitutes an important process in the regulation of CL function. In several species including the buffalo cow, PGF_2α_ functions as a physiological luteolysin that curtails CL function at the end of non-pregnant cycle and prior to parturition [23-26]. Despite its central role in luteolysis, PGF_2α_ actions on CL leading to decrease in P_4_ secretion and subsequent apoptotic changes have not been clearly elucidated. In rats, it is well documented that the initial decrease in luteal function that occurs post PGF_2α_ treatment is precipitated by an increase in P_4_ metabolism i.e. P_4_ gets converted to its inactive metabolite 20α-OHP rather than a decrease in its synthesis [9]. The stimulatory effect of PGF_2α_ on 20α-HSD expression in the CL tissue is well recognised in rodents [27-30]. In ruminants including the buffalo cow, PGF_2α_ causes marked rapid decline in circulating concentration of P_4_ (unpublished data from the laboratory, Davis et al., 2010). As the initial actions of PGF_2α_ on the CL are not well defined, it became of interest to examine whether PGF_2α_ treatment in buffalo cows during luteal phase leads to formation of inactive metabolite such as 20α-OHP. Since the CL of ruminants unlike rodents express P_4_ receptors, it can be argued that perhaps initial decline in P_4_ that occurs in response to PGF_2α_ treatment leads to changes in expression of genes associated with control of luteal function [23,31-33].

In order to determine whether rapid decline in circulating P_4_ was due to its conversion to inactive metabolites, present studies were carried out to examine the activity of 20α-HSD during induced luteolysis in buffalo cows. The results of the present studies demonstrate expression of 20α-HSD in CL and other tissues of the buffalo cow. The importance of 20α-HSD expression in tissues such as spleen, brain and liver is unclear but may be associated with steroid metabolism [18]. Furthermore, despite the increased expression of 20α-HSD post PGF_2α_ treatment, its enzyme activity remained low in the CL during PGF_2α_ treatment. Also, circulating concentration of 20α-OHP did not increase post PGF_2α_ treatment. It is not clear why an increased expression of 20α-HSD was not associated with its increased translation and activity post PGF_2α_ treatment. One explanation could be that PGF_2α_ treatment was detrimental to translational machinery. None the less, the results taken together indicate that decreased circulating P_4_ concentration seen in response to the luteolytic dose of PGF_2α_ treatment does not appear to be the result of metabolism of P_4_ in buffalo cows. The present observation of lack of change in 20α-OHP concentration in response to PGF_2α_ treatment in buffalo cows is in contrast to results reported in rodents by others [3,7,8] and as observed in the present rat studies.

In species such as rodents that do not express classical P_4_ receptors in CL, it becomes of interest to examine whether fall in P_4_ concentration that occurs due to catabolism is sufficient and necessary for initiation of process of luteolysis. Also, the regulation of 20α-HSD expression has to be taken into consideration during PGF_2α_-mediated actions on the luteal tissue. It has been shown that prolactin regulates 20α-HSD expression and inhibition of prolactin secretion results in rapid rise in 20α-HSD expression [34-37]. Whether prolactin has a role in the regulation of 20α-HSD expression and whether PGF_2α_ influences prolactin signaling or other factors in the regulation of 20α-HSD need to be investigated. However, it should be pointed out that few studies carried out employing targeted deletion of 20α-HSD in mice model seems to suggest a minor role for catabolism of P_4_ in the CL [5]. Further, it has been suggested that 20α-HSD may have an important role in the regulation of P_4_ levels in the placenta for growth and development of foetus rather than regulating P_4_ levels systemically [1,2,5].

Several studies have suggested participation of Nur77 during parturition process as well as after exogenous PGF_2α_ treatment [3,7,8]. In the present study, a rapid induction of Nur77 expression in CL in response to PGF_2α_ treatment in buffalo cows was also observed. In mice, studies have been carried out extensively to demonstrate that Nur77 binds to the promoter region of 20α-HSD leading to increased transcription [8]. The participation of Nur77 in the regulation of expression of other steroidogenic genes such as adrenal 21-hydroxylase [38], ovarian 3β-HSD [39], 20α-HSD and aromatase as well as StAR, CYP11A1 and CYP17 genes have been reported [11,12]. In addition to transcriptional activation of 20α-HSD expression, Nur77 has been implicated in thymocytic apoptosis following activation of MAP kinases particularly JNK, p38, and possibly ERK5 [40]. The PGF_2α_-induced luteolysis appears to be initiated through activation of phospholipase C. Earlier reports have suggested a lack of direct participation of PKC during the luteolytic process, but increased intracellular Ca^+2^ and activation of ERK pathway by Nur77 have been suggested to be involved in the PGF_2α_-mediated actions in the rat CL [41,42]. Incidentally, it should be pointed out that several MAP kinases are activated during PGF_2α_-induced luteolysis in the CL of buffalo cows [23] and involvement of MAP kinase pathways have been implicated in the induction of Nur77 expression [40,43]. The above observations point to a critical role of Nur77 in the activation of apoptotic pathway. In the present study, the observation of increased expression of Nur77 suggests that it may be associated with activation of apoptotic pathway, and this is further supported by the observation of increased JAK and p38 activity in CL from buffalo cows treated with PGF_2α_[23]. However, it remains to be determined what role, if any, Nur77 has in pathways/molecules associated with rapid fall in P_4_. Also, whether Nur77 is responsible for increased expression of 20α-HSD remains to be determined.

## Conclusions

In conclusion, studies carried out to examine 20α-HSD expression and circulating 20α-OHP levels in the buffalo cow indicated expression of 20α-HSD in the CL and it transiently increased at 3 and 18 h post PGF_2α_ treatment, but this was not accompanied by increased activity of 20α-HSD. The results also indicated that Nur77, the transcription factor that has been implicated in transcriptional increase of 20α-HSD expression in rodents was also transiently increased in the buffalo cow CL post PGF_2α_ treatment. The results taken together suggest that catabolism of P_4_ does not occur in cattle post PGF_2α_ treatment.

## Competing interests

The authors declare that they have no competing interests.

## Authors’ contributions

TS and RM participated in designing, conducting experiments, analysis of results and preparation of manuscript. KA participated in the preparation of manuscript. All authors have read and approved the final manuscript.
